# Spontaneous Afferent Activity Carves Olfactory Circuits

**DOI:** 10.3389/fncel.2021.637536

**Published:** 2021-03-09

**Authors:** Nelly Redolfi, Claudia Lodovichi

**Affiliations:** ^1^Department of Biomedical Sciences, University of Padua, Padua, Italy; ^2^Neuroscience Institute CNR, Padua, Italy; ^3^Veneto Institute of Molecular Medicine, Padua, Italy; ^4^Padova Neuroscience Center, University of Padua, Padua, Italy

**Keywords:** activity, spontaneous, development, topography, plasticity, olfaction

## Abstract

Electrical activity has a key role in shaping neuronal circuits during development. In most sensory modalities, early in development, internally generated spontaneous activity sculpts the initial layout of neuronal wiring. With the maturation of the sense organs, the system relies more on sensory-evoked electrical activity. Stimuli-driven neuronal discharge is required for the transformation of immature circuits in the specific patterns of neuronal connectivity that subserve normal brain function. The olfactory system (OS) differs from this organizational plan. Despite the important role of odorant receptors (ORs) in shaping olfactory topography, odor-evoked activity does not have a prominent role in refining neuronal wiring. On the contrary, afferent spontaneous discharge is required to achieve and maintain the specific diagram of connectivity that defines the topography of the olfactory bulb (OB). Here, we provide an overview of the development of olfactory topography, with a focus on the role of afferent spontaneous discharge in the formation and maintenance of the specific synaptic contacts that result in the topographic organization of the OB.

## Introduction

The specificity of connections in the nervous system is essential for normal brain function. In most sensory systems, peripheral neurons project axons in specific loci of the brain to form topographic maps that plot out, in a systematically ordered manner, information of the external world. A topographic map is a spatially ordered projection of the sensory surface to higher brain regions. This ordered neuronal organization encodes and integrates essential features of sensory and motor inputs (Kaas, 1997; Cang and Felheim, 2013). The development and maintenance of the architecture of topographic maps is a complex phenomenon regulated by molecules expressed in a specific spatio-temporal pattern and by electrical activity (Zhang and Poo, 2001; Huberman et al., 2008). Molecular cues are thought to govern the first coarse layout of synaptic connectivity that is subsequently refined by distinct forms of electrical activity, in different stages of development. This vision, according to which different regulators, i.e., molecules and activity, act in distinct phases of development, has been modified to accommodate evidence indicating that molecules and neuronal activity interact to define the structure of neuronal circuits, in all developmental stages (Katz and Shatz, 1996; Huberman et al., 2008; Kutsarova et al., 2017).

Electrical activity can be divided into spontaneous and evoked. Unlike evoked activity, which is triggered by sensory inputs (Zhang and Poo, 2001), spontaneous discharges are intrinsically generated and are independent of external stimuli (Fatt and Katz, 1952).

The spontaneous activity emerges during embryonic life when evoked activity is largely precluded. In the very early stages of development, neurons and neuronal precursors exhibit spontaneous activity, which at this stage, occurs mostly at a single-cell level and is not correlated among cells within the neuronal population. This early form of activity affects several neuronal developmental processes such as proliferation (LoTurco et al., 1995), migration (Komuro and Rakic, 1996), and differentiation (Gu and Spitzer, 1995; Spitzer, 2006). As neurons begin to assemble in neuronal circuits, the spontaneous activity becomes correlated among groups of neighbor cells. This patterned neuronal activity has been observed in several brain regions where it serves several functions, such as the formation of central pattern generators (Marder and Rehm, 2005), axon pathfinding in the spinal cord (Hanson et al., 2008), maturation of synapses (Gonzalez-Islas and Wenner, 2006). In particular, it has been shown to inform neuronal circuit development in the developing brain (Garaschuk et al., 2000; Zhang and Poo, 2001; Watt et al., 2009; Malyshevskaya et al., 2013; Kirkby et al., 2013).

In postnatal life, upon maturation of the sense organs, sensory-driven activity refines and consolidates the existing circuits. This remodeling of neuronal circuits takes place during specific postnatal periods, indicated as critical periods, in which circuits are particularly plastic, i.e., able to change synaptic strength and connectivity in response to sensory experience (Hubel and Wiesel, 1970; Hench, 2004).

Intrinsically generated activity persists in the adult brain in most animals and in humans. It appears as spontaneous fluctuations of neuronal activity within local and across wide brain regions creating functional networks that change upon different brain states and cognitive activities (Fox and Raichle, 2007; de Pasquale et al., 2010; Deco and Corbetta, 2011; Leopold and Maier, 2012; Raichle, 2015; Gutierrez-Barragan et al., 2019). Alterations of these neuronal activity fluctuations and therefore alterations in the functional connectivity appear to be correlated to different neurological diseases (Uhlhaas and Singer, 2006, 2010; Palop and Mucke, 2010, 2016). Although there is a growing interest in spontaneous activity, the function and the mechanisms underlying spontaneous oscillations of neuronal networks in adulthood, remain largely to be understood (Schoenfeld et al., 1985; Krishnan et al., 2018).

### Spontaneous Electrical Activity and Sensory Modalities

The role of spontaneous activity in the formation of sensory systems well emerged during the study of the development of the visual system. The pioneering work by Galli and Maffei (1998) demonstrated that spontaneous firing was present in retinal ganglion cells (RGCs) in the retina of rat fetuses. In this context, action potentials could not be triggered by visual stimuli, since the retina is still immature, but emerged from the ability of RGCs to generate spontaneous discharge that could play a key role in shaping circuit formation between the retina and central brain areas. This seminal work inspired several researchers to investigate the pattern and the function of the spontaneous discharge of RGCs, the output neurons of the retina, on the development of the visual system. It was found that neighboring RGCs fire nearly synchronous bursts of action potentials that are separated by long periods of silence. This correlated activity consists of waves of depolarization that sweep across the retina and was therefore indicated as retinal waves. RGC correlated activity is a strong signature of the developing retina and has been found in several vertebrates (Meister et al., 1991; Wong et al., 1993; Wong, 1999; Firth et al., 2005; Torborg and Feller, 2005; Feller, 2009; Ackman et al., 2012). In mice, retinal waves have been observed in the first days after birth and persist till eye-opening, around postnatal day 13–14 (P 13–14). During this period, disrupting RGC correlated activity by pharmacological (Stellwagen and Shatz, 2002; Chandrasekaran et al., 2005) or genetic (McLaughlin et al., 2003; Cang et al., 2005; Ackman et al., 2012) interventions lead to altered RGC projections to the target area, indicating that retinal waves exert an instructive role in defining RGC axonal segregation in eye-specific layers in the lateral geniculate nucleus (LGN). Whether propagation of retinal waves is instrumental also for the development of retinotopy remains more equivocal, although evidence suggested that retinal waves are instructive also for retinotopy (Xu et al., 2015; Arroyo and Feller, 2016; Thompson et al., 2017; but see also Gruss et al., 2003). Retinal waves do not remain confined to the retina but propagate to the lateral geniculate nucleus (LGN; Mooney et al., 1996; Weliky and Katz, 1999) and to the visual cortex (Hanganu et al., 2006; Ackman et al., 2012; Siegel et al., 2012), where they contribute to the generation of a correlated pattern of activity among local neurons. Disrupting correlated RGC activity pharmacologically or genetically was shown to disrupt not only the retinogeniculate projections (see above) but also the connections between the LGN and the visual cortex (Stryker and Harris, 1986; Weliky and Katz, 1999; Chiu and Weliky, 2001; Cang et al., 2005; Ackman et al., 2012; but see also Miller et al., 1989; Crowley and Katz, 1999). These findings unveiled that waves of correlated spontaneous activity among neighbor retinal cells spread across the retina and ascend to neurons located in higher visual areas to direct and refine neuronal wiring in the visual system (Weliky and Katz, 1999; Huberman et al., 2008; Colonnese and Khazipov, 2010; Ackman et al., 2012; Siegel et al., 2012). How can patterns of spontaneous activity direct and refine specific synaptic connections? Theoretical models and experimental evidence seem to indicate that the configuration of the mature structure of neuronal connectivity requires correlated patterns of activity in the presynaptic terminals to match with the patterns of activity in the postsynaptic cells. Furthermore, the presynaptic inputs have to be reinforced by the postsynaptic target to transform correlated patterns of activity in the refinement of neuronal structures. These features reflect Hebb’s principle according to which “neurons that fire together, wire together.” This form of Hebbian activity-dependent plasticity appears to be a plausible mechanistic link between patterns of spontaneous activity and remodeling of synapses in the developing visual system. Indeed, during the developmental period in which the visual system is sensitive to spontaneous activity, retinogeniculate and retinocollicular synapses have been found to show LTP and LTD (Butts et al., 2007; Shah and Crair, 2008). Hebbian form of synaptic plasticity implies an activity-dependent competition among the presynaptic terminals. Only those axonal arbors whose activity correlates with the activity of the postsynaptic target make stable synapses, while those axonal arbors that do not exhibit correlated activity with the postsynaptic cells withdraw and are ultimately eliminated (Butts et al., 2007; Shah and Crair, 2008). As the visual system matures and the animal acquires the ability to process visual stimuli, the visual system relies less on spontaneous activity and becomes increasingly dependent on sensory-evoked activity. It is, however, worth noticing, that even upon maturation of the visual system, spontaneous activity, although with different patterns, persists in the visual system, and continues to contribute to activity-dependent plasticity, that is required to elaborate the existing circuits. For instance, afferent spontaneous activity is required for the effect of neurotrophins on visual cortical plasticity, in a classical paradigm of monocular deprivation. If spontaneous activity is abolished with intraocular injection of TTX, neurotrophins lose their efficacy in counteracting the effects of monocular deprivation in the primary visual cortex (Berardi et al., 1999; Caleo et al., 1999).

The results obtained in the visual system prompted several subsequent studies that aimed at dissecting the role of spontaneous electrical activity in the development of other sensory modalities (Imaizumi et al., 2018) such as the auditory system (Tritsch et al., 2007; Clause et al., 2014; Wang and Bergles, 2014; Müller et al., 2019) or the somatosensory system (Yang et al., 2013; Mitrukhina et al., 2015; Mizuno et al., 2018; Antón-Bolaños et al., 2019). Whether spontaneous electrical activity could shape neuronal circuitry in the olfactory system (OS) remained unknown for a long time. In this review article, we focus on the role of afferent spontaneous activity in the formation and maintenance of the topographic organization of the main olfactory bulb (OB), the first olfactory brain area, in rodents, namely rats and mice.

### The Olfactory System

In rodents, namely rats and mice, odors are perceived by olfactory sensory neurons (OSNs), that constantly regenerate throughout the life of the individual (Graziadei and Graziadei, 1979). They are bipolar neurons with a single apical dendrite that reaches up to the surface of the olfactory epithelium (OE) and an unmyelinated axon that projects directly to the OB. The apical dendrite ends in a “knob,” from which several cilia depart (Firestein, 2001). Odorant receptors (ORs) are expressed on the cilia (Buck and Axel, 1991), and each OSN expresses exclusively a single type of odor receptor in a repertoire of more than 1,000 ORs, in mice (Chess et al., 1994; Malnic et al., 1999).

The OR is a G protein-coupled receptor that upon binding odors, activates a stimulatory olfactory-specific G protein, G_olf_ (Jones and Reed, 1989) which in turn activates adenylyl cyclase type III (ACIII; Bakalyar and Reed, 1990), leading to a rise in cyclic AMP (cAMP). cAMP in turn binds the cyclic nucleotide-gated (CNG) channels, allowing an influx of Na^+^ and Ca^2+^. The calcium ions entering through the CNG channels bind and activate Ca^2+^ dependent-chloride channels causing an efflux of Cl^−^ ions. This intracellular signaling cascade leads to OSNs depolarization to generate action potentials (Kleene and Gesteland, 1991; Firestein, 2001).

Among the elements that characterize the structure of the olfactory epithelium, the identity of the OR and the OR derived cAMP exert a key role in neuronal wiring, acting both at a molecular and a functional level, as we will explain in the following sections.

The OS is a sophisticated system endowed with the inordinate ability to discriminate thousands of different odors. Each OR can recognize specific structural features of the odor molecules (odotopes). Since each odor presents several odotopes, a single odor molecule can activate different types of ORs, with different affinity. In turn, an OR, recognizing distinct odotopes, can bind several odors. In this way, the mammalian OS uses a “combinatorial code” that permits the identification of a myriad of different odorants (Malnic et al., 1999; Mori et al., 1999).

### Topographic Organization of the Olfactory System

#### The Main Olfactory Bulb

In most sensory modalities, nearby receptor neurons in the periphery project to nearby neurons in the brain. Therefore, the spatial relation among receptor neurons is maintained among neurons located in central brain areas. This spatial segregation of sensory afferents provides a topographic map that encodes the quality and intensity of sensory stimuli. The topographic organization of the OS differs from this pattern of neuronal connectivity, in several ways (Murthy, 2011; Fritzsch et al., 2019).

**Figure 1:**
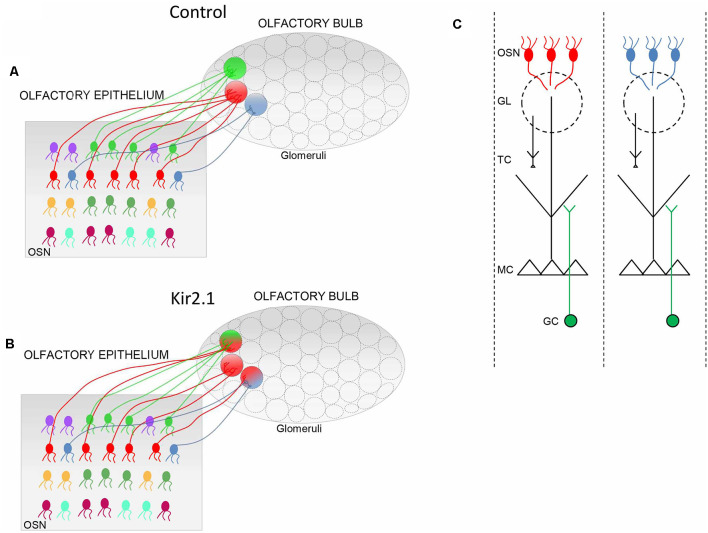
Schematic of the connectivity between the olfactory epithelium (OE) and the olfactory bulb (OB). **(A,B)** In controls, **(A)** olfactory sensory neurons (OSNs) expressing the same odorant receptor (OR; i.e., same color) project their axons in specific loci of the OB to form the corresponding glomeruli (circle with a single color). In Kir2.1 mice **(B)** OSNs expressing the same OR (same colors) project their axons not only to the main homogeneous glomerulus (circle with a single color) but also to multiple additional heterogeneous glomeruli (indicated by a circle with two colors). **(C)** Schematic of odor columns. OSNs expressing the same OR form synapses with the postsynaptic cells, namely the mitral and tufted (M/T) cells, along with the periglomerular cells at the glomerular level. Within the external plexiform layer, M/T cells form synapses with the granule cells. Each glomerulus defines, therefore, a functional unit, indicated as an odor column that processes the sensory information related to a given OR. GL, glomerulus; GC, granule cells.

In the OE only a coarse topography is present. OSNs expressing the same OR are randomly intermixed in different (Ressler et al., 1993; Vassar et al., 1993) but overlapping zones (Miyamichi et al., 2005) along the dorso-ventral axis of the OE. Spatial order is achieved in the OB, where projections of OSNs expressing the same OR, converge to form glomeruli in specific loci on the medial and the lateral side of each OB (Figure 1; Ressler et al., 1993; Vassar et al., 1993; Mombaerts et al., 1996). Glomeruli are spherical structures of neuropil formed by OSNs axons that form synapses with the postsynaptic elements i.e., mitral cells (MC) and tufted cells (TC), along with the periglomerular cells. It is worth noticing that MCs and TCs extend their apical dendrite only in a given glomerulus, contributing to the formation of a cellular column that processes odor information conveyed by the OR expressed by the OSN axons forming the same glomerulus. Therefore, each OSN is associated with a single OR, and the corresponding glomerulus is associated with the same OR, and so they complement postsynaptic cells (MCs and TCs) that extend their apical dendrite exclusively into that glomerulus. In this way, a glomerulus defines a functional unit, also named as odor column, that processes sensory information related to a given OR (Shepherd, 2004).

The spatial segregation of olfactory sensory afferents provides the topographic map of the OB that defines the quality and intensity of olfactory stimuli. As a consequence of this organizational plan, an odor is encoded by a spatial pattern of activated glomeruli (Rubin and Katz, 1999; Uchida et al., 2000; Wachowiak et al., 2000; Belluscio and Katz, 2001). Therefore, disruption of the specific architecture that underlies the topography of the bulb leads to altered odor coding (Fleischmann et al., 2008; Lorenzon et al., 2015).

In line with the logic underpinning olfactory topography, mature glomeruli are formed exclusively by fibers expressing the same OR, indicated as “homogeneous” glomeruli. This distinctive glomerular structure is the result of a refinement process. Indeed, in the early stage of development, OSNs expressing the same OR project to a restricted area of the OB and form multiple glomeruli. Each of these glomeruli is formed by axons expressing different ORs (heterogeneous glomerulus). The process of maturation proceeds during the postnatal period and glomeruli appear like discrete homogeneous units, i.e., formed exclusively by OSNs fibers expressing the same OR (Royal and Key, 1999; Treloar et al., 1999; Potter et al., 2001). Noteworthy, glomerular maturation progresses along with different time courses according to the OR expressed by the OSNs. P2 glomeruli are mature soon after birth (Royal and Key, 1999). M71 glomeruli undergo a prolonged maturation and the number of glomeruli per bulb does not reach the number of glomeruli found in adulthood until P60. On the contrary, the number of M72 glomeruli per bulb reaches the adult level by P20 (Zou et al., 2004).

Similarly, MC, the principal output neurons of the OB, undergo a refinement process related to their dendritic arborization. In the early stage of development MCs exhibit several apical dendrites that terminate in multiple glomeruli. Within the first postnatal week, the supernumerary dendrites withdraw and MCs present the prototypical features characterized by a single primary dendrite with a well-developed tuft extended into a single glomerulus (Santacana et al., 1992; Malun and Brunjes, 1996).

#### The Accessory Olfactory Bulb

A different organization is present in the accessory olfactory system (AOS), specialized in detecting information from pheromones which convey information related to the social communication between individuals (Halpern, 1987; Tirindelli et al., 2009). It is composed by the vomeronasal organ (VNO) of Jacobson in which are located specialized sensory neurons that project to the accessory olfactory bulb (AOB). In turn, the output neurons of the accessory bulb send fibers to distinct brain areas including the medial amygdala and the ventromedial hypothalamus (Halpern, 1987). The organization of the vomeronasal sensory afferents differs from those present in the main OB (Belluscio et al., 1999; Hammen et al., 2014). In 1999, Belluscio and colleagues, employing a gene-targeting strategy to visualize the pattern of sensory neuron projections from VNO to the AOB, showed that neurons expressing a given pheromone OR project to multiple glomeruli (20–30), clustered within broad but spatially restricted domains within the AOB. Moreover, individual glomeruli may receive inputs from more than one type of sensory neuron, in accord with their size. The presence of a small number of distinct domains in the AOB may indicate that the vomeronasal system elicits only a limited array of behavior, in contrast with the main OS in which the activation of specific combinations of discrete glomeruli encode a myriad of different odors and their concentration. The functional organization of the AOB was investigated in-depth also in a recent study. Calcium imaging experiments performed in *ex vivo* preparation of the AOS, in response to urine or sulfated steroids presentation, showed a modular and non-chemotopic spatial organization in the AOB, where individual glomeruli can be tuned to odorants with different molecular features (Hammen et al., 2014).

### The Second Level of Topography in the Olfactory Bulb

**Figure 2:**
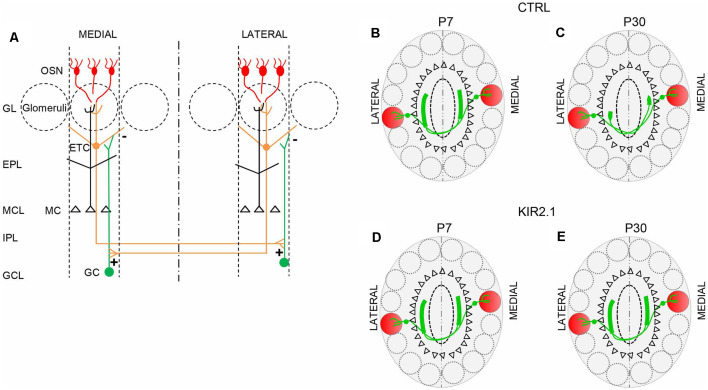
The connection between isofunctional glomeruli. **(A)** Schematic of the intrabulbar link between homologous glomeruli. External tufted cells (ETC) connected to a given glomerulus form excitatory synapses (+) onto the dendrites of the granule cells (GC) in a restricted region of the internal plexiform layer on the opposite side of the olfactory bulb (OB). The GC in turn forms inhibitory synapses (−) on the ETC connected to the homologous glomerulus. This connection is reciprocal. **(B,C)** In control mice, during the early stage of development, ETC related to a given glomerulus, project their axons to a broad area on the opposite side of the OB, beneath, but not limited, to the homologous glomerulus. **(B)** Then, the ETC projection undergoes a refinement process, such that its extension becomes limited to the homologous glomerulus **(C)**. **(D,E)** In mice with reduced afferent spontaneous activity (Kir2.1 mice), ETC’s large projection first formed beneath the homologous glomerulus **(D)** on the opposite side of the bulb, remains larger, assuming the features of unrefined connectivity **(E)**. OSN, olfactory sensory neurons; GL, glomerular layer; EPL, external plexiform layer; MCL, mitral cell layer; IPL, internal plexiform layer; GCL, granule cell layer.

The convergence of like axons to form a glomerulus on the medial side and another glomerulus on the lateral side of each OB results in two mirror-symmetric maps of homologous glomeruli. Compelling evidence indicates that the two symmetric maps are the two halves of an integrated map, not two independent structures (Schoenfeld et al., 1985; Belluscio et al., 2002; Lodovichi et al., 2003). At first, Schoenfeld et al. (1985) performing dye injections in one side of the bulb, observed large projections, related to external tufted cells (ETC), on the opposite side of the OB. Since ORs had not been cloned at the time, it was not possible to establish which structures the ETC projections connected. This open question was addressed several years later by Lodovichi and Belluscio, in Katz’s laboratory. They found that ETCs surrounding a given lateral glomerulus extend their axon on the opposite side of the bulb, forming a projection in the inner plexiform layer, just underneath the medial homologous glomerulus. This connection is reciprocal and creates a single integrated map in which isofunctional odor columns are connected through intrabulbar links (Figure 2). These links are present between homologous glomeruli in the two bulbs of the same animals, and in the bulbs of different animals, providing the second level of topography in the OB (Belluscio et al., 2002; Lodovichi et al., 2003). The role of the intrabulbar link in odor coding remains elusive. Zhou and Belluscio (2008), suggested that ETC projection can shape the OB output through intraglomerular modulation of MC activity. Namely, when ETCs activity precedes olfactory nerve stimulation, the MC response is potentiated, on the contrary, when ETCs activity follows the olfactory nerve stimulation, the MC response is inhibited. However, the functional outcome of this modulatory role and, more in general, the impact of homologous glomeruli on odor coding, remain enigmatic, highlighting the need for further investigations.

From the structure of the topographic organization of the OS emerges that the spatial segregation of OSN afferents hinges on the identity of ORs and not on the spatial relation among OSNs in the periphery, i.e., the nasal epithelium. The tight link between the OR identity and the formation of glomeruli prompted the hypothesis that the OR could have a dual role: it detects odors but also governs OSN projections to the brain. This hypothesis was corroborated by elegant genetic experiments in which mutation in the OR sequence leads to aberrant OSN axons targeting and the formation of a perturbed topographic map (Wang et al., 1998; Feinstein et al., 2004). To act as axon guidance molecules, ORs were proposed to be expressed at the axon terminal, a suitable location for guidance cues. This hypothesis was corroborated by evidence that ORs are expressed locally at the axon terminal (Barnea et al., 2004; Strotmann et al., 2004; Dubacq et al., 2009) where ORs are coupled to local increases of cAMP and Ca^2+^ (Maritan et al., 2009; Pietrobon et al., 2011; Lodovichi and Belluscio, 2012; Movahevi et al., 2016). Moreover, recently, the first putative ligand of the axonal OR has been identified (Zamparo et al., 2019). Altogether, these findings suggest that axonal ORs could act as axon guidance molecules, providing sensory axons with instructions to reach the proper target.

### Role of Odor-Evoked Activity in the Olfactory Map Formation

The evidence that the OR is a key player in the OB topography, prompted the researchers to study the role of odor-evoked activity in the topographic organization of the OB. To study the impact of sensory stimuli on neuronal circuit formation, researchers have extensively exploited the paradigm of sensory deprivation, such as monocular deprivation, for the development of the visual system. This approach cannot be extended to olfaction, since it is not possible to odor-deprive an animal. To circumvent this limitation, a series of genetically modified lines of mice were generated. Each line carries a mutation in one of the molecular components of the intracellular signaling cascade coupled to the OR. This genetic approach has a double advantage: (1) creating anosmic animals; and (2) defining the role of each molecule of the OR signaling pathway in the sensory map formation.

Mice carrying a null mutation in the gene encoding CNG channels, a key molecular determinant of the intracellular signaling pathway coupled to ORs, failed to exhibit responses to odorant stimuli (Brunet et al., 1996). However, axons of OSNs expressing the same OR, coalesce to form glomeruli in the proper location in the OB, as in controls (Lin et al., 2000). Subtle alterations in OSNs convergence were reported only for M72 OR (Zheng et al., 2000). Similarly, the refinement of the mitral cell dendritic tree, although slowed during development, ultimately appears unperturbed, and mitral cells exhibit a single apical dendrite (Lin et al., 2000).

The location of the CNG gene on the X chromosome made it possible to create a cellular mosaic in the OE of CNG mutant females (Zhao and Reed, 2001). In one-half of the cells, the disrupted CNG gene was expressed, resulting in the lack of odor-evoked responses, while in the other half, the wild type gene was transcribed and odor-responsive neurons generated. In this scenario, CNG mutant females exhibited a dramatic loss of OSNs expressing defective CNG channels. This loss was demonstrated to be activity-dependent since OSNs capable of responding to odors (i.e., expressing functional CNG channels) survived, while OSNs lacking CNG functional channels disappeared. This activity-dependent defect was even more evident at the glomerular level. OSNs expressing functional and non-functional CNG channels were able to reach the proper glomerular target, but only those neurons expressing functional CNG channels persisted, while mutant OSN axons were progressively eliminated (Zhao and Reed, 2001). These findings revealed the existence of an activity-dependent competition among OSNs reaching the same target, similar to the one observed in the visual system, such that axons initially project to a common area, but only the active axons persist.

Mice bearing a null mutation in G_olf_, the specific G protein associated with ORs present strikingly reduced electrophysiological responses to odors. However, the convergence of like-axons to form glomeruli is unaltered in these mutant mice (Belluscio et al., 1998).

A completely different scenario was observed in mice carrying a null mutation in adenylyl cyclase III (ACIII), the enzyme required for cAMP synthesis. These mice cannot discriminate several odorants, indicating that cAMP is a critical component of the OR signaling pathway. Furthermore, the OSNs project to the glomerular layer, but they do not form proper glomeruli. As a result of this abnormal organization of sensory afferents, the olfactory map results deeply disrupted (Trinh and Storm, 2003; Dal Col et al., 2007; Zou et al., 2007). An even more severe alteration of OSN projections was observed in another line of mice, in which cAMP synthesis was also abolished. In this line of mice, OSNs expressed a defective OR in which the cytoplasmic end of the transmembrane domain III was mutated, preventing the interaction between ORs and the coupled G protein. This genetic manipulation abolishes cAMP synthesis and OSNs expressing the defective OR never entered the glomerular layer but remained in the olfactory nerve layer (Imai et al., 2006).

Overall, these data indicate that evoked activity *per se* does not affect the convergence of OSN axons to form glomeruli in specific locations in the OB. However, projection and coalescence of OSN axons were deeply affected by any genetic manipulation that abolishes cAMP synthesis, unraveling a critical role of the OR-derived cAMP in the sensory map formation. The lack of effect on the OB topography observed upon abolishing G_olf_ expression, the G protein that activates AC to synthesize cAMP, can be explained by the presence of another G protein (G_sα_), normally predominant in cilia of immature OSNs (Menco et al., 1994), that might be re-expressed upon elimination of G_olf_ expression.

### Spontaneous Afferent Activity Shapes Neuronal Circuits in the Olfactory Bulb

The slight contribution of OR-evoked activity in the formation of the topographic map of the OB induced researchers to explore the possible function of afferent spontaneous activity in the OB topography. In this respect, it is worth noticing that ORs mediate not only odor-evoked activity but also spontaneous firing. This finding is corroborated by the fact that OSNs expressing a defective OR lack spontaneous discharge (Reisert, 2010; Connelly et al., 2013). The spontaneous activation of the OR induced to hypothesize that this mechanism could be at the origin of the OR-derived cAMP, which is required for the sensory map formation (Nakashima et al., 2013). The vast diversity of ORs and the considerable variation in spontaneous activity and in the level of cAMP even among neurons expressing the same OR makes unclear how the specificity of targeting can be achieved in this context.

To analyze the impact of the spontaneous afferent activity on the olfactory map formation, Yu et al. (2004) developed gene-targeting approaches that permit either the conditional inhibition of neurotransmitter release (TeTxLC mice) or the conditional hyperpolarization of OSNs (Kir2.1 mice). In the first transgenic line of mice (TeTxLC mice), the tetanus toxin light chain (TeTxLC), expressed in OSNs, proteolytically cleaves VAMP2, which exerts a key role in neurotransmitter release from synaptic vesicles. To visualize the convergence of like axons, TeTxLC mice were crossed with transgenic lines of mice in which OSN expressing a given OR, such as P2, co-expressed GFP (i.e., P2- GFP mice). It was found that in a non-competitive condition, in which all OSNs express TeTxLC, OSNs axons converged to form glomeruli, as in controls. On the contrary, under competitive conditions, when only a specific subpopulation of neurons (such as neurons expressing the P2-odorant receptor) expressed TeTxLC, these OSNs projected to their glomerular target but failed to maintain proper synaptic connections. This synaptic instability was followed by a striking reduction in the number of OSNs (Yu et al., 2004).

In the second mouse model (Kir2.1 mice), OSNs overexpress the inward rectifying potassium channel Kir2.1 that causes hyperpolarization of the OSN membrane leading to a strong reduction of spontaneous discharge (Yu et al., 2004). By analyzing the development of the glomerular map, Yu et al. (2004) found that in Kir2.1 mice, OSNs axons entered the OB with a significant temporal delay with respect to controls and they converged to form the main glomeruli but they also targeted several additional glomeruli. These findings indicated that spontaneous activity does not instruct the formation of the sensory map, although it is required for the refinement of olfactory synaptic connections.

To widen and deepen the knowledge of the impact of afferent spontaneous activity on olfactory circuit formation, additional investigations were conducted by Lorenzon et al. (2015). At first, they performed a thorough analysis of the effect of Kir2.1 overexpression on the electrical activity of OSNs. They found that Kir 2.1 OSNs exhibit a striking reduction in spontaneous firing, while they maintain unperturbed the ability to respond to odors. These results indicate that Kir2.1 mice are an ideal model to study specifically the role of afferent spontaneous activity on the topography of the OB. The unperturbed response to odors in Kir2.1 OSNs prompted Lorenzon et al. (2015) to analyze odor response in the OB. Performing functional imaging experiments, they found that odor-evoked responses were readily recorded in glomeruli devoid of afferent spontaneous activity, although the functional maps were deeply disrupted. Namely, a given odor activated a higher number of glomeruli and each glomerulus responded to a higher number of odorants than in controls. Moreover, the average size of the activated areas was bigger, while the amplitude of the signal recorded in each glomerulus, in response to odors, was smaller in Kir2.1 in respect to controls.

Since odor-evoked responses in OSNs were similar in mutants and controls, these alterations in the OB functional maps had to be ascribed to aberrant neuronal wiring in the OB. In this respect, by studying OB neuronal connectivity, Lorenzon et al. (2015) found that functional alterations reflected with an exquisite precision abnormality in neuronal wiring in Kir2.1 mice. Indeed, OSNs expressing the same OR, such as P2, projected to several glomeruli, in addition to the main stereotypical glomeruli (Figure 1). These additional glomeruli were heterogeneous and located in the vicinity of the main homogeneous glomeruli. As the topographic map is essential to encode and discriminate odors, not surprisingly Kir2.1 mice exhibited defects in odor discriminations.

Also, Lorenzon et al. (2015) extended their analysis to the second level of the OB topography: the intrabulbar link. At first, they analyzed the development of the intrabulbar projections since previous studies were limited to adults (Belluscio et al., 2002; Lodovichi et al., 2003). They found that in the early stage of development (at postnatal day 7, P7), in wild type (WT) mice, the projection between homologous glomeruli is present but not confined to the homologous glomeruli, but larger. Through a refinement process, the projection becomes limited to the homologous glomeruli, at P30 (Figure 2). In Kir 2.1 mice the intrabulbar projection remains larger in adults as well (Figure 2).

Altogether these findings indicate that spontaneous discharge does not play an instructive role in the formation of the sensory map, but it is required to refine OB synaptic connections. Indeed, in absence of afferent spontaneous activity, neuronal connectivity in the OB remains unrefined, assuming features of immature neuronal circuits. This altered pattern of connectivity, in turn, affects neuronal circuits functional outcome, resulting in impaired odor discrimination. In contrast, no defects were found in the development of the postsynaptic elements of the OB, the MC, that present a single apical dendrite entering in a single glomerulus both in Kir2.1 and control mice.

## Critical Periods

In most sensory modalities, neuronal circuits are shaped by neuronal activity mostly in distinct time windows in early postnatal life, indicated as critical periods. In these periods, neurons exhibit heightened plasticity to reflect changes of the incoming flux of information in modifications of the morphology of dendritic and/or axonal arborization and/or in increase or reduction of the synaptic strength. The time points at which the critical periods begin and end are well defined, although they vary according to the specific neuronal circuits and the animal species. The visual system is the modality where critical periods have been best characterized, since it was the first modality in which they were studied and identified, in the seminal works of Hubel and Wiesel (1970). Today is still the premier model system for critical period plasticity studies. Hubel and Wiesel (1963, 1970) first demonstrated that manipulation of vision, such as monocular deprivation, within a given period of postnatal development, results in the loss of responsiveness to the deprived eye in the primary visual cortex, providing the first evidence of the existence of critical periods. Functional consequences of monocular deprivation appear first and are then followed by anatomical rearrangement of horizontal connections and geniculo-cortical projections (Antonini and Stryker, 1993; Trachtenberg and Stryker, 2001). These results unravel the activity-dependent competition between the two eyes to innervate bands of tissue in layer IV of the primary visual cortex. Based on the timing when disruption of activity affects synaptic maturation, it is possible to identify different windows of heightened plasticity. The “first critical period” in the visual system occurs at the retinogeniculate synapses. Disruption of retinal waves prevents eye-specific lamina formation (Penn et al., 1998; Rossi et al., 2001). The segregation of eye-specific projections in distinct thalamic layers (or patches in mice, that do not exhibit specific layers, in the thalamus) is driven by an activity-dependent competition between the two eyes to control distinct areas of the thalamus as demonstrated by forcing binocular innervation of the tectum, in animals that normally have the tectum innerved exclusively by the contralateral eye (Constantine-Paton and Law, 1978). After the closure of critical periods, synaptic reorganization and remodeling are difficult to induce (Katz and Shatz, 1996; Hench, 2004). Since the seminal works of Hubel and Wiesel (1970), an overwhelming number of studies have dissected the mechanisms underlying the critical periods, identifying molecular and functional factors that regulate heightened plasticity during the critical period, such as the role of neurotrophins as a feedback mechanism that strengthen the synaptic connections (Hübener and Bonhoeffer, 2014), the key role of GABAergic circuit in regulating experience-dependent plasticity in the visual cortex (Hench et al., 1998; Huang et al., 1999; Hench, 2004), the distinct forms of neuronal network plasticity, such as Hebbian and homeostatic plasticity in the visual cortex (Kirkwood and Bear, 1995; Maffei et al., 2006; Maffei and Turrigiano, 2008; Turrigiano, 2017). The works in the visual system prompted to study critical periods in other sensory modalities, such as auditory (Kral, 2013), Somatosensory (O’Leary et al., 1994; Jamann et al., 2018), or motor system (Walton et al., 1992; Huntley, 1997).

## Olfaction I: Critical Periods for the Topographic Organization in the Olfactory Bulb

**Figure 3:**
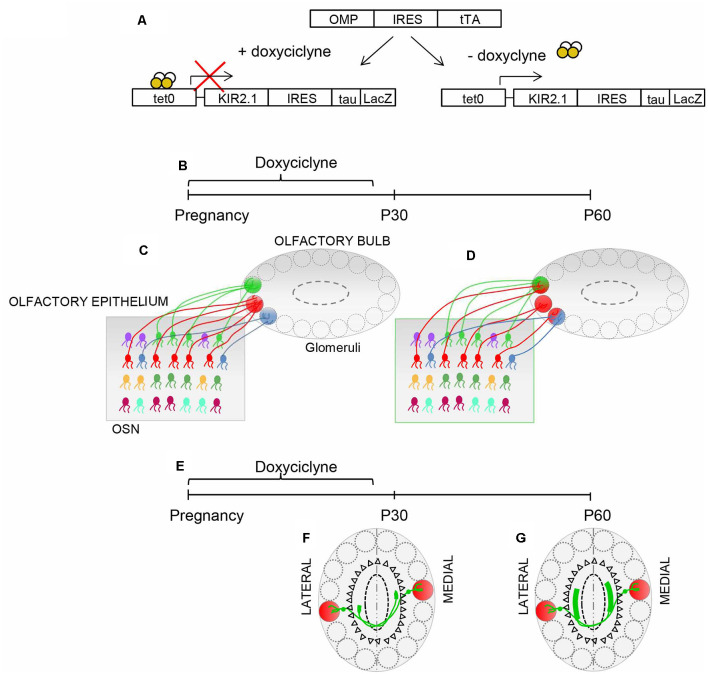
Plasticity in the OB. **(A)** Schematic illustrating the inducible nature of the Kir2.1 construct. Mice carrying OMP-IRES-tTA and tetO-Kir2.1-IRES-tau-lacZ alleles express Kir2.1 and tauLacZ only in the absence of doxycycline. Doxycycline suppresses the expression of Kir 2.1. **(B,E)** Schematic of the experimental strategy: doxycycline is supplied during pregnancy until postnatal day 30 (P30). The organization of connection was analyzed at P30 and P60. **(C)** The specificity of connectivity is present in Kir.2.1 mice at P30, upon suppression of Kir2.1 expression **(D)**. The overexpression of Kir2.1 between P30 and P60 induces regression of the already refined connectivity **(D)**. **(F)** Similarly, homologous glomeruli are linked in a point-to-point manner at P30, upon doxycycline treatment. **(G)** However, the overexpression of Kir2.1 between P30 and P60 leads to unrefined connectivity between the homologous glomeruli.

The presence of critical periods in olfaction is still a matter of debate. Lorenzon et al. (2015) exploited the inducible nature of the Kir2.1 construct to ascertain whether the convergence of sensory neurons may be susceptible to modification of afferent activity. Suppression of Kir2.1 overexpression during gestation, till postnatal day 30 (P30), resulted in a normal sensory map, in the OB at P30. In contrast, overexpression of Kir2.1 between P30 and P60 induced the regression of the already refined connectivity in the olfactory map and in the intrabulbar connections (Figure 3), indicating that activity-dependent synaptic plasticity persists into adulthood.

Although the high degree of plasticity is retained in the mature OS, some works suggested an unconventional critical period for the olfactory map (Ma et al., 2014; Tsai and Barnea, 2014; Wu et al., 2018). Ma et al. (2014) took advantage of Kir2.1mice, where overexpression of Kir2.1 in OSNs is known to result in abnormal OB topography (Yu et al., 2004; Lorenzon et al., 2015). Kir2.1 mice in which ectopic Kir2.1 expression was suppressed (by doxycycline administration) within the first postnatal week, develop a normal olfactory map. However, suppression of overexpression of Kir2.1 in OSNs after the first postnatal week was associated with an abnormal olfactory map.

In parallel, Tsai and Barnea (2014) by using a different strategy, found the presence of an unconventional olfactory critical period, confined to the perinatal period. They found that perinatal expression of ectopic ORs (i.e., MOR 28) lead to the rerouting of like axons to form multiple ectopic glomeruli scattered throughout the bulb, whereas the expression of ectopic receptors, once the sensory map is formed, does not cause OSNs axons rerouting, suggesting a critical period soon after birth, during the formation of the sensory map.

These studies seem to have identified an unconventional critical period confined to the first postnatal week. However, the constant regeneration of OSNs, along with the recurrence in adulthood of the same strategy exploited by OSNs during development, to reach and converge into the OB (Gogos et al., 2000) may hold a key for plasticity throughout adulthood. The different results observed in different studies highlight the need for further investigations to clarify the presence and the extent of critical periods at this level of the OS.

## Olfaction II: Critical Period in the Olfactory Cortex

Critical period plasticity affects different hierarchical levels of sensory and motor systems. Sensory driven modulation of synaptic strength and remodeling of thalamocortical synapses are key features of activity-dependent plasticity during critical periods in several sensory modalities, such as the visual system, where they have been first identified and characterized (Hubel and Wiesel, 1963), somatosensory system (Woolsey and Wann, 1976; Jones, 2000) and auditory system (Zhang et al., 2001). In the olfactory cortex pyramidal neurons receive sensory information directly from the OB, without a thalamic relay. Axons of mitral and tufted cells, bundled into the lateral olfactory tract (LOT), form synapses on the distal dendrites of pyramidal cells in the olfactory cortex (LOT synapses), while associational fibers, originating from different brain areas, make synaptic contacts on the proximal and basal dendrites of pyramidal neurons (ASS synapses). Cindy Poo, in Isaacson laboratory (Poo and Isaacson, 2007), provided evidence for NMDA-receptor dependent plasticity at the sensory synapses (LOT), on the distal dendrites of pyramidal neurons, limited to a brief period of early postnatal development. On the contrary, associational synapses on the same pyramidal cells maintain synaptic plasticity into adulthood. In parallel, they found that dendritic spines at sensory (LOT) synapses mature faster than dendritic spines associated with associational fibers. These data agree with previous results obtained in the same lab (Franks and Isaacson, 2005), where they showed a strong activity-dependent down-regulation of NMDA receptors and a modest increase in AMPA receptors at sensory synapses (LOT) but not at associational synapses (ASS) in pyramidal neurons in olfactory cortex. The loss of activity-dependent plasticity at the sensory synapses, early in development, seems to suggest that coding of olfactory information becomes rapidly “hardwired,” although very early experiences can contribute to remodeling synapses in the olfactory cortex and may provide a mechanism for early imprinting of odor experience. The persistence of plasticity at the associational synapses lay the basis for modifying odor information, such as salience of odors, or more in general any process of odor learning and memory throughout the life of the individuals.

The presence of a critical period in the olfactory cortex in the very first weeks of postnatal life is consistent with the early maturation of olfaction, which can operate at birth. Most animals rely on olfactory cues for their survival in this very early stage of development, when most sensory modalities are still immature (Sullivan, 2003).

## Spontaneous Activity: Origin, Pattern, and Function in Different Sensory Modalities

### In the Visual System

During development, synaptogenesis, maturation, refinement of synaptic contacts, and circuit architecture depend mostly on spontaneous activity (Spitzer, 2006). Although present in multiple brain areas during development, spontaneous discharge exerts distinct functions in different brain regions (Garaschuk et al., 2000; Zhang and Poo, 2001; Marder and Rehm, 2005; Gonzalez-Islas and Wenner, 2006; Hanson et al., 2008; Malyshevskaya et al., 2013). In the visual system, where spontaneous activity has been best characterized, patterns of spontaneous activity, i.e., the retinal waves, span across the retina and ascend to higher visual regions to modulate the activity in these brain areas. Retinal waves appear to be the primary source of patterned activity during the development of the visual system, transferring patterned activity to the thalamus (Mooney et al., 1996; Stellwagen et al., 1999; Ackman and Crair, 2014), and the cortex (Hanganu et al., 2006). In parallel, spontaneous discharge independent of retinal inputs has been identified in the LGN (Weliky and Katz, 1999) and in the primary visual cortex (Siegel et al., 2012). As far as the spontaneous patterns in the LGN, they seem to result from the interaction of the retina, thalamus, and cortical inputs (Weliky and Katz, 1999). Some differences in pattern and interaction of spontaneous activity can be observed in different species (Ackman and Crair, 2014). The complex architecture of the visual system emerges from an activity-dependent competition mechanism, that in line with Hebb’s principle, strengthen and consolidate, pre-postsynaptic cell pairs with correlated activity, and weaken and eliminate uncorrelated pairs. The spatial and temporal features of the retinal waves appear to be required to establish the precise spatial segregation and topographic maps in the visual system (Mooney et al., 1996; Stellwagen et al., 1999; Ackman et al., 2012; Ackman and Crair, 2014). Indeed, disruption of retinal waves results in enlargement of axon arbores and alterations of topographic mapping (Penn et al., 1998; Stellwagen and Shatz, 2002; McLaughlin et al., 2003; Cang et al., 2005; Chandrasekaran et al., 2005; Ackman et al., 2012). Spontaneous activity, therefore, plays a critical role in the formation of the topographic organization of the visual system, in the early phase of development. The propagation of patterned spontaneous activity reflects the spatial organization of the visual system, where neighbor cells in the retina project to neighbor cells in the thalamus, and in turn, neighbor cells in the thalamus project to neighbor cells in the cortex. Correlated activity among neighbor RGCs is therefore instrumental to maintain and transfer the spatial relation-information from RGCs to the higher levels of the visual system. Patterns of spontaneous activity act as a template- matching mechanism to mold the spatial-based architecture of the entire visual system. Retinal waves appear very early in development and last till eyes opening, which occurs around postnatal day 13–14 in mice. During this period, the neuronal circuit of the retina changes, and so the features of the spontaneous patterns of activity (Feller, 1999; Blankenship and Feller, 2010). What is the origin of retinal waves? Early in development, retinal waves are triggered by a class of cholinergic interneurons, the starburst amacrine cells, that act as pacemakers, that depolarize spontaneously (Zhou, 2001; Zheng et al., 2006). As starburst cells are densely interconnected, this spontaneous depolarization easily propagates from one cell to another giving rise to retinal waves. Although spontaneous activity has a critical function in the development of the topographic organization of the visual system, it is not the only determinant. Molecular cues, such as Eph receptors and their ligands ephrin direct axonal projections to their target (Feldheim and O’Leary, 2010). The relation between molecules and activity is complex and not entirely elucidated. Patterns of spontaneous activity could strengthen synaptic connections between coactive cells leading to Ca^2+^ influx that in turn regulates the expression of specific genes encoding proteins that modulate synaptic morphology, function, and organization. The induced genetic program may trigger the reorganization of the synaptic machinery by changing the composition of ion channels, the function of neurotransmitter receptors, the clustering of existing synaptic contacts, or even by leading to the formation of new synapses. The innate genetic program is known to encode for axon guidance cues that govern the first layout of the neuronal connectivity. Activity may also modulate the expression axon guidance cues, and in turn refine the first coarse layout of neuronal connectivity defined first by the innate genetic program.

### In the Olfactory System

The OS offers a different scenario. Though spontaneous activity is present and important for the refinement and maintenance of neuronal circuits in the OB, it does not exert an instructive role in the formation of the OB topography, but rather it has a permissive role. Indeed, disruption of spontaneous activity (Yu et al., 2004; Lorenzon et al., 2015) does not prevent the formation of the main homogeneous glomeruli, but hampers the refinement of the olfactory circuitry, disrupting the structure of the olfactory map with the presence of additional heterogeneous glomeruli.

The different functions of spontaneous discharges seem to reflect the different principles that inform the topographic organization of these two sensory modalities, vision and olfaction. In olfaction, not the spatial continuity among sensory neurons in the periphery, as in a vision, but the identity of the ORs instructs the development of the topography in the OB. In line with these different organizational principles, the molecular determinant, i.e., the ORs, instruct the formation of the map and exert a primary role, while spontaneous activity intervenes in a second phase, to refine the already established topographic map. The spatio-temporal dynamics of the patterns of spontaneous activity are significantly different. Discharges of RGC exhibit a distinct structure, characterized by a burst of depolarization interleaved by long periods of silence, that recur with specific spatial and temporal dynamics (Meister et al., 1991; Feller et al., 1997; Butts et al., 1999; Xu et al., 2016). On the contrary, in the OS, the spontaneous discharge does not appear to have a distinct spatio-temporal characteristic. The origin and propagation of spontaneous activity are completely different in the two systems. In the retina, it emerges from the connectivity among the cellular elements of the retina network and is dictated by the functional properties of the pacemaker-like cells of the retina. In olfaction, it is a single-cell-based phenomenon due to the spontaneous conformational changes of the ORs that result in neuronal depolarization (Reisert, 2010; Connelly et al., 2013). As the spontaneous discharge in the retina originates from the interconnectivity of the retina network, changes in the retina connectivity, as occur during development, result in changes in the patterns of spontaneous activity (Feller, 2012). This evolution of the pattern of activity, during development, is not present in olfaction, since the spontaneous discharge is a cell-autonomous phenomenon related to the spontaneous conformational change of the ORs. Furthermore, in the ORs, the depolarization does not spread to correlate the activity of neighbor cells, since the topography of the bulb is not based on the spatial continuity of cells in the periphery, but on the identity of the receptors, that are scattered in an almost random disposition in the OE; and even cells expressing the same receptors, display a considerable variation of spontaneous activity rate. As the propagation of spontaneous activity to higher brain olfactory areas, very little is known. The topographic organization of the piriform cortex remains elusive, although evidence seems to indicate that the highly organized topography of the bulb is lost in the cortex, which appears devoid of a rigorous topographic organization (Ghosh et al., 2011; Sosulski et al., 2011; Igarashi et al., 2012; Diodato et al., 2016).

A signature of the spontaneous activity in the visual system is that it favors activity-dependent competition between the two eyes for the control of distinct layers in the thalamus and distinct bands of tissue in the primary visual cortex. This form of activity-dependent competition is not present in the OS, since sensory neurons project only homolaterally and the entire complement of neurons expressing a given OR contributes to the formation of the corresponding glomerulus. However, when spontaneous discharge suppression is limited to a subpopulation of neurons expressing a given OR, such as P2, creating a competitive environment in the OSNs population, the convergence of the “silent” sensory neurons is dramatically perturbed (Yu et al., 2004). In particular, when the spontaneous activity is abolished at weaning, once P2-OSN axonal convergence to form the glomeruli has already occurred, these axons devoid of spontaneous discharge fail to maintain the synaptic connection with the target, withdraw and progressively disappear, mimicking the activity-dependent competition among axonal projections during the development of the visual system. To establish a proper model of activity-dependent competition among the OSN population, Cao et al. (2007) devised a genetic strategy to selectively suppress neurotransmission (expressing the tetanus toxin light chain in OSNs) in a random subset of OSNs. This approach allowed the generation and validation of a genetic *in vivo* axonal activity-dependent competition paradigm. A long follow-up confirmed that this paradigm triggered competition among OSN axons and revealed that active axonal neurons were able to establish synapses with the postsynaptic target, while the silent axons exhibited morphological alterations and ultimately degenerated. The authors demonstrated a specific effect of BDNF signaling on axonal terminal-pruning under competitive conditions (Cao et al., 2007). These results, although with some differences, mimic the activity-dependent competition of axonal projections and the effect of neurotrophins on axonal arborization in the activity competitive environment, present during development in the visual systems (see above).

Molecules have a prominent role in the formation of the topography of the OB. However, even in olfaction, the development of the topographic maps is regulated by a complex interplay between molecules and activity, that remains largely to be clarified. In particular, how spontaneous activity could affect the expression of molecules involved in the development of the topography of the bulb remains elusive. Nakashima et al. (2013) suggested that the spontaneous conformational change which undergoes the OR could trigger the synthesis of the OR-derived cAMP, which, in turn, may activate a genetic program, leading to the expression of axon guidance cues, such as neuropilin. In the OS, with more than 1,000 ORs, it is difficult to envision how specificity can be achieved in this model, as even OSNs expressing the same OR exhibit a high variation in the level and pattern of spontaneous activity. A second model proposes that the ORs at the axon terminal is activated by a few molecules expressed in gradients in the OB. The OR- derived cAMP and Ca^2+^ may exert their role locally, directing turning and elongation of the growth cone, and at the nucleus, where they can activate, *via* CREB phosphorylation, genes encoding for axon guidance cues (Imai et al., 2006; Maritan et al., 2009; Pietrobon et al., 2011). The identification of the first putative ligand of the axonal OR (Zamparo et al., 2019), seems to corroborate this second model. The spontaneous activity would act in a subsequent phase, to refine the convergence of OSN axons (Yu et al., 2004; Lorenzon et al., 2015). The role of the OR derived cAMP at the axon terminal (Maritan et al., 2009; Pietrobon et al., 2011) and the nuclear level (Imai et al., 2006; Maritan et al., 2009; Pietrobon et al., 2011) explains why only mutations that abolish cAMP synthesis prevent the formation of the sensory map. These results are in line with an overwhelming number of observations indicating that cAMP and Ca^2+^ exert a key role in elongation and turning of the axon terminal in several systems, acting locally and modulating gene expression of axon guidance cues (Song et al., 1997; Zheng and Poo, 2007).

In conclusion, it emerges that the structure and function of spontaneous activity in the visual and the OS reflects the different structural and functional organization of the two sensory modalities, although in both systems spontaneous discharge exerts an important role in remodeling neuronal connections.

## Conclusions

Neuronal firing exerts a key role in several aspects of the development of the central nervous system. Early in development, innate spontaneous activity directs sensory projections to their target. These first patterns of connectivity are then further elaborated in postnatal life when the interaction with the environment exerts a crucial role in reshaping neuronal connectivity. Olfaction differs from this organizational plan in several ways. Spontaneous activity exerts an important role in the early phase of development, before sense organs are operative, although it acts in different ways in different systems such as the olfactory and the visual system. These differences are likely to reflect the specificity of the architecture of each sensory modality and the principles that inform their topographic organization. In the visual system, the distinct features of the photonic signals are parsed by different cells located in specific locations of the retina. The visual space is mapped onto the retina. This spatial distribution is then maintained through the hierarchy of the visual system. Similarly, features of somatosensory stimuli (Chen-Bee et al., 2012; Harding-Forrester and Feldman, 2018), or sounds (Knudsen and Konishi, 1978; Brodal, 1981; Schreiner and Cynader, 1984; Schreiner and Mendelson, 1990; Schreiner and Winer, 2007) can be easily represented in a continuous distribution that reflects the continuity of the somatic space or transduces in spatial continuity the low-high frequency range of sounds, from the receptor sheets throughout higher brain areas. Patterns of correlated activity among neighbor cells implement this spatial organization at each level. It is worth noticing that several complex circuits, such as ocular dominance column and orientation selectivity, are formed at birth or before eye-opening, highlighting the critical role of a spontaneous pattern of activity in shaping these circuits (Rakic, 1976; Crair et al., 1998; Crowley and Katz, 2000).

As for olfactory stimuli, represented by a myriad of small organic molecules, that differ in shape, size, charge, pressure, mapping in space appears an unsuitable solution. As a consequence, OSNs are deployed almost randomly in the OE, reflecting the volatile essence of olfactory stimuli. The necessity to have a code, a map, to identify odors emerges in the OB as “a construction of the brain,” that assembles receptors of the same identity together, that represents the only rule that governs the organizational plan of the OS, resulting in a “discrete” sensory map. Where the discrete unit is identified by the identity of the odorant receptor itself. Which other factor could have been more appropriate to represent more than 1,000 ORs? So why does the OR have such a prominent role, but odor-evoked activity has not a significant effect on the OB topography? We can envision several explanations: (1) olfaction is almost completely mature at birth. Most animals rely on olfactory cues for their survival, including human infants. Babies at birth cannot see well, but they recognize their mother by the smell; and this is true for most mammals. Olfaction is critical to creating attachments between parents and progeny (Leon, 1992; Sullivan, 2003). (2) In contrast to sounds or lights that can be decomposed in a few universal features, such as colors, intensity, directionality, location, odors are very different, and there are not a few common features that could instruct the map. In other words, if odors could drive the development of the topographic maps, we will likely not have an olfactory topographic map, defined as an invariant organization of neuronal circuits among animals. Since according to the type of odors a single animal is surrounded by, the map would be organized differently. Spontaneous discharge retains the ability to refine and maintain the circuits, although in a permissive way, highlighting again the importance of the ORs in defining the convergence and the location of OSNs to form the glomeruli. The importance of the OR is dictated also by the fact that even the pattern of spontaneous activity is regulated by the OR, by its spontaneous conformational change. Therefore, the OR acts at two distinct levels: molecular and functional. Although critical, the OR is not the only determinant. We favor a model in which axonal ORs act as axon guidance molecules and are activated by complementary ligands expressed in the OB. The recent identification of the first putative ligand of the axonal ORs seems to corroborate this hypothesis (Zamparo et al., 2019). The OR-ligand interaction defines the target areas where OSNs expressing a given OR project. This first layout of the olfactory connectivity is then refined by the basal activity of OSNs, which is regulated again by the OR identity. A complex interplay among ORs, other molecular cues (Cho et al., 2009), and activity carve the final configuration of the OB topography.

Olfaction differs from other sensory modalities also concerning the existence of a critical period. The constant regeneration of OSNs that continuously reform with exquisite precision stereotyped connections with the target favors a model in which plasticity persists throughout life. The evidence seems to corroborate this vision (Gogos et al., 2000; Lorenzon et al., 2015). However, in other studies, an unconventional critical period has been identified in the first postnatal week. The mixed evidence of a critical period along with the high degree of plasticity in the OS highlight the need for further investigations to clarify this point.

As for the connection between the bulb and the cortex, a critical period has been identified in the very early stage of development for sensory inputs, suggesting that this circuit becomes “hard-wired” pretty early (Franks and Isaacson, 2005; Poo and Isaacson, 2007). The coding of the odors seems to be established mostly by molecular cues also at the cortical level. In contrast, associational fibers retain synaptic plasticity that is critical to encoding the salience of odor stimuli and for odor learning and memory (Franks and Isaacson, 2005; Poo and Isaacson, 2007).

Activity, either spontaneous or evoked, is mostly related to activity-dependent competition between sensory afferents for the control of the target areas. Even in this respect olfaction differs from other sensory modalities since most fibers project unilaterally and converge on structure dedicated to neurons expressing the same features (i.e., the same OR). However, when the competition is forced, olfactory afferents with different levels of activity mimic the behavior observed in the visual system, based on competitive interactions (Yu et al., 2004; Cao et al., 2007; Lorenzon et al., 2015).

Altogether the multiple roles of the OR identity in olfactory topography unravel the elegant way in which Nature created order from an almost random distribution of sensory neurons in the periphery. ORs, as guidance cues, direct axons to their glomerular target, while ORs as the origin of spontaneous activity, refine and maintain neuronal connectivity.

## Author Contributions

NR and CL designed the review. NR wrote the first draft. CL revised and contributed to the final version of the review. All authors contributed to the article and approved the submitted version.

## Conflict of Interest

The authors declare that the research was conducted in the absence of any commercial or financial relationships that could be construed as a potential conflict of interest.
